# Bile acids impact the microbiota, host, and *C. difficile* dynamics providing insight into mechanisms of efficacy of FMTs and microbiota-focused therapeutics

**DOI:** 10.1080/19490976.2024.2393766

**Published:** 2024-09-03

**Authors:** Arthur S. McMillan, Casey M. Theriot

**Affiliations:** a Genetics Program, Department of Biological Sciences, College of Science, North Carolina State University, Raleigh, NC, USA; b Department of Population Health and Pathobiology, College of Veterinary Medicine, North Carolina State University, Raleigh, NC, USA

**Keywords:** *Clostridioides difficile*, recurrent CDI, microbiota, bile acids, fecal microbiota transplantation, nuclear receptors

## Abstract

*Clostridioides difficile* is a major nosocomial pathogen, causing significant morbidity and mortality worldwide. Antibiotic usage, a major risk factor for *Clostridioides difficile* infection (CDI), disrupts the gut microbiota, allowing *C. difficile* to proliferate and cause infection, and can often lead to recurrent CDI (rCDI). Fecal microbiota transplantation (FMT) and live biotherapeutic products (LBPs) have emerged as effective treatments for rCDI and aim to restore colonization resistance provided by a healthy gut microbiota. However, much is still unknown about the mechanisms mediating their success. Bile acids, extensively modified by gut microbes, affect *C. difficile*’s germination, growth, and toxin production while also shaping the gut microbiota and influencing host immune responses. Additionally, microbial interactions, such as nutrient competition and cross-feeding, contribute to colonization resistance against *C. difficile* and may contribute to the success of microbiota-focused therapeutics. Bile acids as well as other microbial mediated interactions could have implications for other diseases being treated with microbiota-focused therapeutics. This review focuses on the intricate interplay between bile acid modifications, microbial ecology, and host responses with a focus on *C. difficile*, hoping to shed light on how to move forward with the development of new microbiota mediated therapeutic strategies to combat rCDI and other intestinal diseases.

## Introduction

*Clostridioides difficile* is a Gram-positive, spore-forming, anaerobic pathogen that causes *C. difficile* infection (CDI), a major healthcare-associated infection with significant morbidity and mortality worldwide. It is classified as an urgent threat by the Centers for Disease Control and Prevention (CDC), based on gaps in research and treatment despite aggressive actions being taken.^1^ In 2017, there were approximately 462,100 cases and 20,500 deaths related to CDI.^2^ Approximately, half of these cases were classified as community-associated as opposed to healthcare-associated, although community-associated CDI represents only about a fifth of in-hospital deaths.^2^

A major risk factor for CDI is antibiotic usage, which kills protective gut microbes that are able to provide colonization resistance.^3,4^ The standard of care for patients with CDI is treatment with vancomycin or fidaxomicin, with a preference for fidaxomicin due to its preservation of the gut microbiota and reduced incidence of recurrence.^5^ Treatment with vancomycin and to a much lesser degree fidaxomicin continues to alter the gut microbiota allowing *C. difficile* to reestablish infection, leading to recurrent CDI (rCDI) in approximately 25% of patients after initial treatment.^3,6^ The definition for rCDI is recurrence of diarrhea and a positive *C. difficile* test within 8 weeks of completing CDI directed therapy.^7^ The risk of recurrence can increase by 20–25% for subsequent recurrences.^8,9^ Fecal microbiota transplantation (FMT), a treatment derived from healthy donor stool, has emerged as an effective therapy for rCDI, with cure rates exceeding 90%.^6^ The goal of this therapy is to restore colonization resistance that is lost after antibiotic treatment.

Recently, the FDA approved the first microbiota-derived therapeutics for the treatment of rCDI. These microbiota-focused therapeutics source healthy stool in such a way as to reduce the risk of introducing harmful bacteria and aim to standardize the processing of stool for FMT.^10,11^ Products such as Rebyota and VOWST are derived from healthy donor stool, with VOWST having an additional ethanol purification to select for spore forming bacteria.^10,11^ Live biotherapeutic products (LBPs) composed of bacterial consortia, such as MET-2 and VE303, are also being investigated.^12,13^ As of February 2024, MET-2 is in phase 1 clinical trials for rCDI and ulcerative colitis (UC), while VE303 is in phase 2 clinical trials for rCDI. However, the specific mechanisms underlying the efficacy of FMTs and LBPs as well as the mechanisms through which gut microbes provide colonization resistance are still an area of active investigation.

**Figure 1.**
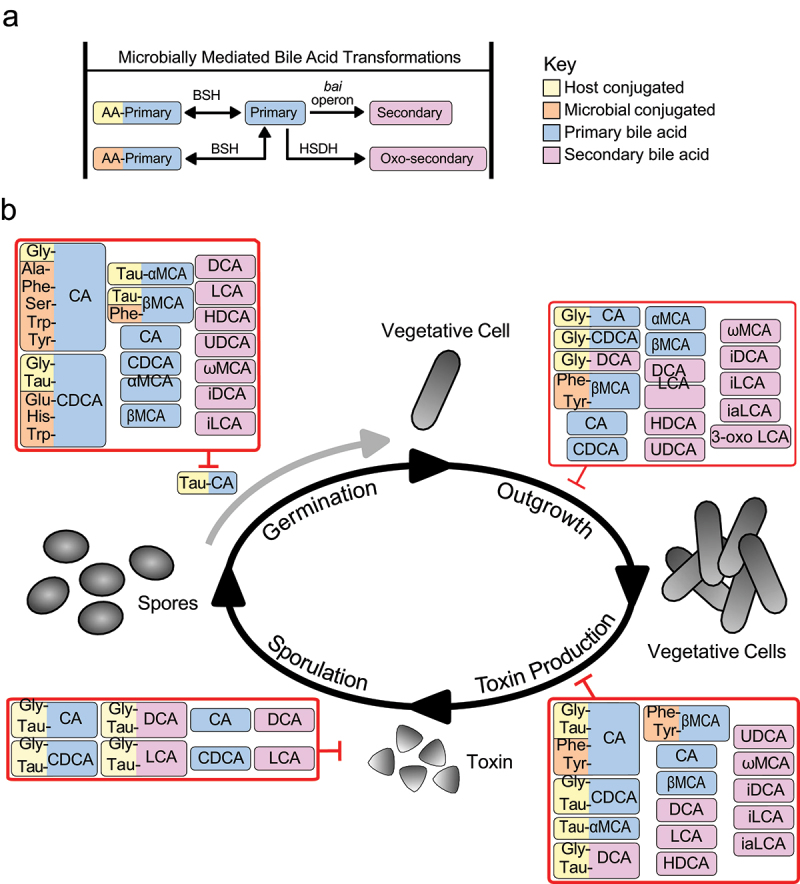
The gut microbiota modifies bile acids which are able to inhibit different stages of the *C. difficile* life cycle. (a) Basic overview of the main bile acid altering enzymes discussed in this review. BSHs deconjugate and reconjugate bile acids with an amino acid or directly exchange the conjugated amino acid for another. Amino acids can be host or microbial conjugated. The *bai* operon removes a 7α-hydroxyl group producing secondary bile acids. HSDHs dehydroxylate bile acids resulting in an oxo-bile acid, in this case a oxo-secondary bile acid. (b) Effect of bile acids on different stages of the *C. difficile* life cycle *in vitro*. TCA mediated spore germination of *C. difficile* spores (grey arrow)^14–16^ is inhibited by various bile acids (red box).^17,18^ outgrowth of vegetative *C. difficile* is impacted by a variety of bile acids (red box).^17,18^ the production of toxin is inhibited by bile acids (red box) through reducing expression of toxin or toxin activity.^15,17,19^ bile acids also bind directly to *C. difficile* toxin, reducing its toxicity in the host (red box).^21^ abbreviations: AA, amino acid; Ala, alanine; BSH, bile salt hydrolase; *bai*, bile acid inducible; CA, cholate; CDCA, chenodeoxycholate; DCA, deoxycholate; glu, glutamate; gly, glycine; HDCA, hyodeoxycholate; his, histidine; HSDH, hydroxysteroid dehydrogenase; iDCA, isodeoxycholate; iaLCA, isoallolithocholate; iLCA, isolithocholate; LCA, lithocholate; phe, phenylalanine; ser, serine; tau, taurine; trp, tryptophan; tyr, Tyrosine; UDCA, ursodeoxycholate; αMCA, α-muricholate; βMCA, β-muricholate; ωMCA, ω-muricholate; 3-oxo LCA, 3-oxolithocholate.

A proposed mechanism that we hypothesize contributes to the efficacy of microbial-based therapies is microbiota mediated alterations in the gut bile acid pool (Figure 1(a)), which impacts *C. difficile*, the microbiota, and the host. Bile acids exert antimicrobial effects against *C. difficile* and other members of the gut microbiota through different mechanisms such as disruption of the bacterial cell membrane.^4,22,23^ Microbial-derived secondary bile acids are able to inhibit different stages of the *C. difficile* life cycle, such as spore germination, vegetative outgrowth, toxin expression, production, and activity^4,17,19,22,24,25^ (Figure 1b). Bile acids also impact the host immune response through interactions with nuclear receptors like farnesoid X receptor (FXR), Takeda G-protein receptor 5 (TGR5), pregnane X receptor (PXR), and retinoic acid-related orphan receptor γT (RORγT).^26^ Bile acid-induced activation of these receptors plays a role in immunity, specifically the differentiation of Th_17_ and T_reg_ cells.^27^

There are also non-bile acid mediated microbial interactions through which the gut microbiota may protect against *C. difficile* colonization, including the production of metabolites and exchange of nutrients between *C. difficile* and members of the gut microbiota. Other metabolites, such as short-chain fatty acids (SCFAs), including acetate, propionate, and butyrate return after FMT.^28^ Butyrate inhibits *C. difficile* growth and promotes epithelial barrier integrity and modulates the host immune responses.^29–31^ Butyrate was also found to increase *C. difficile* toxin production and sporulation.^31^ Additionally, many members of the gut microbiota are able to compete for nutrients, such as amino acids important for Stickland fermentation with *C. difficile*.^32^ Whereas others like *Enterococcus* are able to provide *C. difficile* with nutrients it requires for growth, including ornithine.^33^

In this review, we will focus on how bile acid modifications carried out by members of the gut microbiota impact the pathogen, microbiome, and host response with a lens on *C. difficile*. We will also consider the role of competition of nutrients and metabolite production by the microbiota. We will highlight more recent work that provides evidence for how members of the gut microbiota maybe contributing to the efficacy of FMTs and LBPs for the treatment of rCDI and other intestinal diseases.

## Bile acids and *C. difficile*

Bile acids are synthesized from cholesterol in the liver, conjugated with an amino acid, stored in the gallbladder, and secreted when the host eats a meal.^34^ Their role during digestion is to act as surfactants, allowing for the absorption of dietary fats and vitamins. Once they reach the terminal ileum, ~95% of bile acids are reabsorbed through enterohepatic circulation.^35^ Gut bacteria modify bile acids during intestinal transit, thereby changing the chemistry and diversity of the bile acid pool. Modifications made by the microbiota include deconjugation of the conjugated amino acid, reconjugation or a swap of the conjugated amino acid, dehydrogenation, dehydroxylation, and epimerization. Changes made to the sterol core, rather than the conjugated amino acid, mark the conversion of a primary bile acid to a secondary bile acid. In-depth reviews that explore bile acid modifications in humans and animals can be seen here.^36,37^

Various bile acids directly impact multiple stages of the *C. difficile* life cycle *in vitro* (Figure 1(b)). Bile acids such as taurocholate (TCA) are germinants of *C. difficile* spores^14−^34], while other bile acids such as chenodeoxycholate (CDCA) inhibit spore germination^14,15,17,22,38^ (Figure 1b). Microbially derived secondary bile acids are able to inhibit *C. difficile* spore germination, vegetative cell growth, and toxin activity^17,19,20^ (Figure 1b). The mechanisms through which bile acids inhibit growth are attributed to their antimicrobial and detergent-like properties, which change based on how they are modified.^34^ *C. difficile* is exquisitely sensitive to many of these modifications. Additionally, conjugated and unconjugated forms of the primary bile acids, cholate (CA) and CDCA, as well as secondary bile acids, deoxycholate (DCA) and lithocholate (LCA), can bind to *C. difficile* toxin TcdB mitigating its cell toxicity^21^ (Figure 1(b)). The combined repetitive oligopeptide (CROP) region of TcdB is essential for bile acid binding, which causes major conformational changes when bound.^21^ Several reviews have previously detailed the relationship between *C. difficile* and the bile acid pool and its relevance in colonization resistance here.^3,25,39–43^

Bile acids that are modified by the gut microbiota are associated with successful FMTs, potentially indicating their importance in rCDI (Table 1).^18,28,54,55^ The gut microbiota encodes a variety of bile acid altering enzymes that act on either the sterol core or the amino acid conjugated to it, including, but not limited to, bile salt hydrolases (BSHs), hydroxysteroid dehydrogenases (HSDHs), and those in the *bile acid inducible* (*bai*) operon (Figure 1(a)). These modifications and specifically how they help or hinder *C. difficile* are discussed in more detail below.

**Table 1.** Efficacy of FMT, microbiota-focused therapeutics, and LBPs on rCDI and their impact on the bile acid pool and the microbiome.

Intervention	Impact on rCDI	Impact on bile acid pool	Impact on microbiome	References
FMT	81–93% prevention of *C. difficile* recurrence in human studies.	Reduction in primary bile acids including TCA, GCA, CA, TCDCA, GCDCA, CA. Increase in secondary bile acids including DCA, LCA, and HDCA. Reduction in primary MCBAs, loss of histidine, serine, and valine conjugates. Increase in secondary MCBAs, increase in glutamate, tryptophan, and tyrosine conjugates.	Increase in alpha diversity. Decreases in Enterobacteriaceae, Lactobacillaceae, Enterococcaceae, Veillonellaceae, and Megaspharaceae. Increases in Lachnospiraceae, Acutalibacteraceae, Ruminococcaceae, Oscillospiraceae, Anaeroboracaceae, Butyricicoccaceae, CAG-74, Bacteroidaceae, Rikenellaceae, Bifidobacteriaceae, Coriobacteriaceae, and Eggerthellaceae.	6,18,28,44–47
Rebyota	88% prevention of *C. difficile* recurrence in phase 3 clinical trial.	Significant reduction in TCA, GCA, and CA. Significant increase in DCA and LCA.^48^ Reduction in conjugated and deconjugated primary bile acids. Increase in secondary bile acids, particularly deconjugated.	Increase in alpha diversity. Decreases in Gammaproteobacteria and Bacilli. Increases in Bacteroidia and Clostridia.	48–50
VOWST	87.1% prevention of *C. difficile* recurrence in phase 3 clinical trial	Increase in secondary bile acids compared to placebo. Increase in LCA and DCA correlates with engraftment of VOWST-based microbes.	Engraftment of enriched taxa, Clostridia and Erysipelotrichia, observed after treatment with VOWST. Significant increase in abundance of other organisms alongside VOWST-based microbes, namely Bacteroidetes.	51,52
VE303	86.2% prevention of *C. difficile* recurrence in phase 2 clinical trial	Bile acid abundances return to pre-vancomycin treatment levels in healthy volunteers. Increase in deconjugated secondary bile acids coupled with decrease in conjugated primary bile acids.	VE303 strains displaced native Clostridia including *Blautia spp*. and *Erysipelotrichaceae bacterium 21_3.* Expedited return of native gut microbiota after cessation of antibiotics in healthy volunteers.	13,53
MET-2	79% prevention of *C. difficile* recurrence in phase 1 clinical trial	Not evaluated.	Increase in alpha diversity after treatment with MET-2. Increase in Lachnospiraceae, Bifidobacteriaceae, Ruminococcaceae, Erysipelotrichaceae, and Coriobacteriaceae. Decrease in Enterobacteriaceae, Lactobacillaceae, Streptococcaceae, and Fusobacteriaceae.	12

## Bile salt hydrolases (BSHs)

BSHs are encoded by a variety of microbial Phyla including Bacillota, Bacteroidota, Actinomycetota, and Euryarchaeota.^56^ BSHs are traditionally known to cleave host-conjugated amino acids, either glycine or taurine, from conjugated bile acids.^57^ This first step is often a prerequisite for other bile acid modifications.^57^ The simple conversion of conjugated bile acids to deconjugated bile acids increases the inhibition of *C. difficile* vegetative growth, and the bile acid is more inhibitory as the conversions get more complex.^15,58^

Historically, it was proposed that the purpose of microbial BSHs was to detoxify conjugated bile acids, thereby providing a fitness benefit for colonization in the harsh gut environment by the microbes that encode them.^59^ Additionally, it was suggested that BSHs provide nutrients in the form of liberated amino acids (either taurine or glycine) for the microbes that encode them or the surrounding gut microbiota.^60^ However, recent studies leveraging both Gram-positive *Lactobacillus* and Gram-negative *Bacteroides* found that bile acids become more toxic to bacteria after deconjugation.^61,62^ *Lactobacillus gasseri* and *Lactobacillus acidophilus* each have a taurine-specific BSH and a glycine-specific BSH.^61^ Knockouts of these BSHs resulted in increased growth of these strains *in vitro* in the presence of certain bile acids including conjugated forms of CA, particularly in *L. gasseri*.^61^ Utilizing these knockout strains, the production of deconjugated bile acids by these BSHs decreased the membrane integrity of both microbes.^61^ BSH knockouts of *L. gasseri* and *L. acidophilus* also outcompeted their wild-type counterparts in gnotobiotic mouse models.^61^ BSH knockout strains of *Bacteroides thetaiotaomicron* behave similarly. In a recent paper, different combinations of *B. thetaiotaomicron*’s bile acid altering enzymes, including two BSHs and one HSDH, were knocked out, and the resulting phenotypes were evaluated.^62^ BSH knockouts of *B. thetaiotaomicron* showed an increase in growth in the presence of different bile acids, particularly in conjugated forms of DCA, when compared to wildtype.^62^ BSH knockout strains of *B. thetaiotaomicron* also showed broad changes in their transcriptome including carbohydrate, amino acid, lipid, and energy metabolism when compared to wildtype.^62^ Counter to the dogma that BSHs liberate amino acids to use as a nutrient, *B. thetaiotaomicron* altered the expression of its polysaccharide utilization loci (PULs) in the presence of bile acids rather than amino acid metabolism.^62^ The specific PUL that changes in expression was dependent on the bile acid *B. thetaiotaomicron* encountered.^62^ Most bile acids increased expression of a starch utilization PUL in *B. thetaiotaomicron*, while the secondary bile acid DCA had the broadest impact by activating PULs involved in the degradation of α-mannans, host and dietary glycans, mucins, rhamnogalacturonan I and II, and starch.^62^ These findings indicate that BSH mediated changes to bile acids modify the toxicity of bile acids as well as signaling bacteria to utilize different nutrients in the gut.

Recent work has indicated that BSHs may play an additional role in the production of bile acids conjugated with a variety of non-canonical amino acids, outside of glycine and taurine, collectively referred to as microbially conjugated bile acids (MCBAs) or bacterial bile acid amidates (BBAAs).^15,63,64^ A screen of 70 bacterial species identified 27 different species across multiple Phyla capable of conjugating amino acids to -CA, -CDCA, or -DCA.^65^ A recent survey of 654 unique BSHs from the microbiota in the Integrated Gene Catalog identified a selectivity loop that dictates substrate preference of BSHs in multiple taxa.^15^ The ‘G-X-G’ motif within the selectivity loop prefers taurine conjugated bile acids while the ‘S-R-X’ motif prefers glycine conjugated bile acids.^15^ Further characterization of electrostatic interactions of the selectivity loop implicates the ‘Y-S-R-G’ motif as a preference for aromatic MCBAs.^66^ A taurine-specific lactobacilli BSH cocktail was given to mice in a CDI model and was able to inhibit spore germination and growth of *C. difficile* in the small and large intestines. This was associated with the deconjugation of primary bile acids as well as the production of primary MCBAs (i.e., MCBAs with a primary bile acid sterol core).^15^ These primary MCBAs, specifically alanine, phenylalanine, serine, tryptophan, and tyrosine conjugated CA (Ala/Phe/Ser/Trp/Tyr-CA), glutamate, histidine, tryptophan conjugated CDCA (Glu/His/Trp-CDCA), and phenylalanine-βMCA (Phe-βMCA) inhibit TCA mediated spore germination^15^ (Figure 1b). *C. difficile* vegetative growth was inhibited by Tyr-βMCA and Phe-βMCA.^15^ MCBAs also inhibit the expression of TcdA, specifically phenylalanine, and tyrosine conjugated CA (Phe/Tyr-CA) as well as phenylalanine and tyrosine conjugated βMCA (Phe/Tyr-βMCA).^15^ Host conjugated primary and secondary bile acids also impact different stages of the *C. difficile* lifecycle as seen in Figure 1(b).

Bacteria encoding BSHs are significantly associated with MCBA production, and most bacterial isolates that produce at least one MCBA have a *bsh* homologue.^63^ Unconjugated bile acids also upregulate transcription of *bsh* genes in a strain known to produce MCBAs, *Bifidobacterium longum* NCTC 11,818.^63^ BSH inhibitors were able to significantly decrease the production of MCBAs by *B. longum in vitro* and biochemical assays with the purified BSH encoded by this organism showed both acyltransferase (i.e., TCA to AA-CA) and reconjugation activity (i.e., CA to AA-CA).^63^ This novel activity of BSHs was also confirmed in a strain of *Bacteroides fragilis* using genetic knockouts and complementation, confirming this activity occurs in BSHs encoded across Actinomycetota and Bacteroidota.^63^ In newborn infants, the production of MCBAs increased as their microbiota developed from birth to 1 year and positively correlated with colonization of *bsh* encoding bacteria.^63^

In a different study, *Clostridium perfringens* BSH also demonstrated acyltransferase activity and reconjugation activity.^64^ This BSH produced MCBAs conjugated with most amino acids except for aspartate and proline.^64^ Further, *in vitro* analysis determined 19 of 29 strains of bacteria across Actinomycetia, Verrucomicrobia, Gammaproteobacteria, Bacilli, and Clostridia produced at least one MCBA.^64^ This activity was especially prevalent in the Lachnospiraceae Family.^44,64^ Hierarchical clustering of bacterial conjugation profiles indicated little association between phylogeny of these organisms and BSH activity.^64^ Sequence analysis indicated amino acid substitution at position 82 in the active site of BSHs, separate from the selectivity loop, may dictate the diversity of amino acids that BSHs can conjugate to bile acids to produce MCBAs.^64^ There is evidence that MCBAs enter enterohepatic circulation and therefore should have an opportunity to interact with a variety of host receptors.^64^ Though MCBA concentrations are lower than liver conjugated bile acids in healthy individuals, MCBAs have been observed in concentrations equal to or higher than primary bile acids in the stool of human bariatric surgery patients.^64,67^ In human bariatric surgery, a patient’s total MCBA pool concentration was approximately 77.7 μM compared to the total primary conjugated and secondary bile acid pool concentration of approximately 34.2 μM.^64^

Primary MCBAs, are also enriched in patients with inflammatory bowel disease (IBD), and cystic fibrosis.^68,69^ In IBD, there is no change in the level of secondary MCBAs (i.e., MCBAs with a secondary bile acid sterol core). This is in contrast to rCDI patients before and after FMT, where there is a shift from high primary MCBAs pre-FMT to high secondary MCBAs post-FMT.^44^ The BSHs in highest abundance post-FMT were encoded by many members of the Lachnospiraceae Family.^44^

Differences have been observed in how *C. difficile*, members of the gut microbiota, and the host respond to bile acids conjugated with taurine, as opposed to glycine, and now to non-canonical amino acids.^15,19,61,62^ In light of recent discoveries that challenge conventional wisdom, the precise role of BSHs encoded by the gut microbiota is an area of new investigation and could have major implications for the design of microbiota-focused therapeutics.

## Hydroxysteroid dehydrogenases (HSDHs)

HSDHs that act on bile acids are encoded by a variety of organisms including Bacillota, Pseudomonadota, Bacteroidota, Actinomycetota, and Archaea.^44,70,71^ HSDHs convert hydroxyl groups to ketones at positions 3, 7, or 12 in either the α or β orientation, and 7α-HSDHs are the most studied.^72,73^ Although they can be components of a larger pathway, such as the *bai* operon, these enzymes are also found independently in some organisms.^74^ In some cases, HSDHs have activity on conjugated bile acids.^62^ It is hypothesized that the keto bile acids produced by HSDHs are further modified to produce epimers or remove the hydroxyl group, and thus are often referred to as intermediates. Recently, advances in mass spectrometry have allowed for the detection of these keto bile acids and epimers in urine and serum, indicating these bile acids enter enterohepatic circulation and may be interacting with host receptors.^75^

Epimers of LCA arising from modifications of HSDHs, including 3-oxo LCA, isoLCA (iLCA), alloLCA (aLCA), 3-oxo aLCA, and isoalloLCA (iaLCA) have been found to be enriched in the stool of centenarians, implicating their role in sustained health.^20^ These bile acids are bactericidal and cause damage similar to β-lactam antibiotics.^20^ These bile acids are also able to decrease carriage of *C. difficile* and other Gram-positive pathogens *in vivo*.^20^ Colonization of a prolific producer of these epimers, Odoribacteraceae St21, significantly increased fecal iaLCA levels while reducing *C. difficile* shedding to non-detectable levels in a mouse model genetically modified to have similar bile acid profiles to humans.^20^

*In vitro* studies have shown that 3-oxo LCA, iLCA, and iaLCA effectively inhibit the growth of *C. difficile*^19,20^ (Figure 1(b)). As each modification is made, there is a decrease in minimum inhibitory concentration (MIC) of *C. difficile*. While CDCA exhibits an MIC of 1.25 mM, this value drops to 0.1 mM with the conversion to 3-oxo LCA, and even further to 0.03 mM and 0.02 mM with the subsequent conversions to iLCA and iaLCA, respectively.^19^ Even at subinhibitory concentrations, iLCA and iaLCA significantly reduce toxin expression by *C. difficile*.^19^ Moreover, these bile acids, along with their precursor LCA, diminish the toxin activity of *C. difficile in vitro*.^19^ iLCA and iaLCA also exhibit a more pronounced impact on the growth of *C. difficile* compared to other commensal gut microbes, particularly Gram-negatives, while causing minimal effects on host cell viability.^19^

HSDHs can also function on cholesterol-derived steroids. Gut microbe-encoded 3β-HSDH can degrade estradiol and testosterone, leading to depression in patients.^76,77^ Encoding HSDH provides a range of fitness benefits to microbes, including detoxifying bile acids and being able to use bile acids as electron donors for the electron transport chain.^72,78,79^ In patients undergoing FMT for rCDI, significantly higher levels of HSDH-modified bile acids such as 3-oxo LCA, iLCA, and 12-oxo LCA were observed post-FMT compared to pre-FMT.^44^ Random Forest Analysis showed that these bile acids were among the most significantly different and important metabolites.^44^ As our understanding of HSDHs evolves, these enzymes continue to be crucial for altering cholesterol-derived biomolecules in the gut.

## The bile acid inducible (*bai*) operon

The *bai* operon is encoded by <1% of metagenome-assembled genomes (MAGs) identified in the gut of healthy humans.^80^ Bacillota, specifically Ruminococcaceae and Lachnospiraceae, are the most abundant *bai* operon-encoding bacteria.^80,81^ The *bai* operon comprises a series of genes involved in the removal of the 7α-hydroxyl group from bile acids. This operon is comprised of six core genes that carry out 7α-dehydroxylation: *baiB, baiA, baiCD, baiE, baiF*, and *baiH*, as well as *baiG* which encodes a transporter to bring bile acids into the cell.^82,83^ Other less characterized genes associated with this operon have also been described in some organisms including *baiI, baiN, baiJ, baiK, baiL, baiM*, and *baiP*.^82,84–86^ The removal of the 7α-hydroxyl group results in the conversion of unconjugated primary bile acids, such as CA and CDCA, into the secondary bile acids DCA and LCA, respectively. Recent findings have identified some of the accessory genes associated with the *bai* operon, namely *baiJ* and *baiP*, as having an important role in the 7α-dehydroxylation of CDCA.^87^ It is known that organisms encoding the *bai* operon carry out 7α-dehydroxylation, however there are still many unknowns regarding the regulation and the substrate specificity of the different enzymes in this operon.^88^

Antibiotic-induced changes to the microbiota can modulate colonization resistance against *C. difficile* in mice and in humans. Often, these changes are due to the loss of 7⍺-dehydroxylating bacteria such as *Clostridium scindens* ATCC 35704, which has been associated with resistance to *C. difficile* in a secondary bile acid-dependent manner.^89^ Additionally, *C. scindens* ATCC 35704 has been shown to inhibit CDI in patients undergoing hematopoietic stem cell therapy, the radiation from which alters the gut microbiota in a way similar to antibiotics.^89^ While antibiotics alter the structure of the gut microbiota, they also alter the gut metabolome. Alongside antibiotic-induced decreases in secondary bile acids, glucose, free fatty acids, and dipeptides, there are increases in primary bile acids, amino acids, and sugar alcohols.^4^ The metabolites that increased post antibiotics in mice were able to support *C. difficile* TCA-mediated spore germination and vegetative growth with amino acids *C. difficile* is auxotrophic for, and sugars including mannitol, fructose, sorbitol, raffinose, and stachyose *in vitro*.^4^ These findings were further confirmed in a mouse model where broad-spectrum antibiotics resulted in a significant loss of secondary bile acids and members of the Lachnospiraceae and Ruminococcaceae, and an increase in *C. difficile* germination and outgrowth *ex vivo*.^22^

The inhibition of multiple stages of the *C. difficile* life cycle by secondary bile acids has been reviewed previously.^25,40^ Briefly, secondary bile acids produced by the *bai* operon including DCA, iDCA, LCA, iLCA, and hyodeoxycholate (HDCA) were found to inhibit TCA-mediated spore germination, growth, and toxin activity in several clinically relevant strains of *C. difficile in vitro*^17,22^ (Figure 1(b)). Work has also been done to determine whether *bai-*encoding bacteria can produce sufficient quantities of secondary bile acids to inhibit *C. difficile in vitro*. Four strains of Lachnospiraceae, *C. scindens* VPI 12708, *C. scindens* ATCC 35704, *Clostridium hiranonis* TO-931, and *Clostridium hylemonae* TN-271 were characterized for their ability to inhibit *C. difficile* in a series of *in vitro* assays.^90^ While *C. difficile* outcompeted these strains in co-culture, supernatants of both *C. scindens* strains grown in CA were able to produce enough DCA to inhibit *C. difficile* growth.^90^ This was associated with CA induced increased expression of the *bai* genes in strains of *C. scindens*, and this was not observed with supernatants of *C. hiranonis* or *C. hylemonae* grown in CA.^90^

In human studies, *bai* operon genes are found in significantly higher abundance in patients without CDI than those with CDI.^91^ Secondary bile acid profiles are also distinct in CDI patients, which have low secondary bile acid abundance compared to non-colonized or asymptomatically colonized *C. difficile* patients, which had higher levels of secondary bile acids.^92^ Post-FMT samples in patients being treated for rCDI had a significant increase in *baiA* genes, primarily those encoded by the Lachnospiraceae Family.^44^ Post-FMT samples also had a significant increase in secondary bile acids, specifically DCA, LCA, HDCA, epideoxycholate (EDCA), 3-oxo LCA, iLCA, and 12-oxo LCA.^44^ Of these secondary bile acids that returned post-FMT, targeted metabolomics identified the return of many MCBAs with DCA as the sterol core (Table 1).^44^ Evaluating the abundance of other secondary bile acid amidates (i.e., MCBAs with a secondary bile acid core), such as those with an LCA sterol core, is still difficult due to the lack of synthesized standards.

Microbiota-focused therapeutics have also been observed to restore the secondary bile acid pool. rCDI patients treated with Rebyota showed a shift from dominance of primary bile acids to secondary bile acids, specifically driven by an increase in DCA and LCA (Table 1).^93^ Many spore-forming bacteria encode the *bai* operon, highlighting its role in products that select for spores such as VOWST or communities of spore formers such as VE303. Secondary bile acids and the bacteria that produce them play an important role in restoring colonization resistance against CDI, and it is important that these organisms and the enzymes they encode are further biochemically characterized.

## Bile acids and the gut microbiota

Inhibition of growth by bile acids is not unique to *C. difficile*. Primary and secondary bile acids also exert varying effects on microbial growth across different taxa. As stated previously, bile acids inhibit bacteria by disrupting their membrane integrity.^4,22,23^ However, the concentration of bile acid required to disrupt membrane integrity can vary greatly by species. For example, glycocholate (GCA), which is generated by the host, has a MIC of 20 mM for *B. thetaiotaomicron, Lactobacillus acidophilus, Lactobacillus gasseri*, and *Staphylococcus aureus*.^61,62,94^ However, once GCA is converted to CA by BSHs, the MIC changes to 10 mM, 5 mM, 2.5 mM, and 20 mM, respectively.^61,62,94^ When CA is converted to DCA, via 7α-dehydroxylating bacteria, the MIC changes again to 0.625 mM, 10 mM, 10 mM, and 1 mM, respectively.^61,62,94^ The largest difference in MIC among the strains listed here was observed in the 7α-dehydroxylation of CA to DCA. This variation in response to secondary bile acids highlights how small changes in the bile acid pool can impact the fitness of members of the microbiome.

A recent study using gnotobiotic mice used a consortium of over 100 strains, denoted hCom1a, to demonstrate nuances of niche establishment with and without *C. scindens* ATCC 35704, and *C. hylemonae* DSM 15053, the only microbes in this consortium that perform 7α-dehydroxylation. By simply removing these two organisms, >100-fold changes in the relative abundance of eight strains in the consortia were observed.^95^ The removal of the 7α-dehydroxylating strains not only resulted in the elimination of 7α-dehydroxylated bile acids, but also an increase in other modifications to the 7-hydroxyl groups such as dehydrogenation and epimerization.^95^ The taxonomic shift associated with this was very specific, with decreases in *Ruminococcus, Roseburia*, and *Eubacteria* and increases in *Veillonella, Clostridium, and Dorea*. These changes appear to be species specific, as many other organisms at the same genus level in this consortium were not affected to such a degree.

Investigating genes involved in 7α-dehydroxylation, namely the *bai* operon, has been difficult due to the lack of genetic tools for the microbes that encode it. However, recent work has been able to clone a *bai* operon into *C. sporogenes* and determined a minimal gene set required to perform 7α-dehydroxylation.^82^ This allowed for characterization of each enzyme in the reductive arm of 7α-dehydroxylation.^82^ This opens up the possibility for future studies to include strains with the appropriate background as a control, rather than removing 7α-dehydroxylating bacteria or using closely related species.

In fact, most bile acids directly modify the structure of the gut microbial community. Mice exogenously fed host-derived primary bile acids, TCA and GCA, had large changes to the microbial composition of their gut, marked by a significant decrease in the abundance of *Verrucomicrobia*.^96^ In addition, mice fed the microbial-derived secondary bile acid UDCA had a significant increase in Lachnospiraceae as well as increases in Lactobacillus, Clostridiales, and unclassified Bacillota alongside decreases in Ruminococcaceae.^97^ The administration of bile acids to mice significantly influenced both the bile acid pool and the synthesis of host bile acids.^96,98^ Bile acids given therapeutically to humans are also associated with changes to the microbiota. Patients with colorectal adenomatous polyps treated with UDCA had significant shifts in their microbial community composition, marked by an increase in *Faecalibacterium prausnitzii* coupled with a decrease in *Ruminococcus gnavus*.^99^

MCBAs can also differentially impact the microbiota. A prolific MCBA producer *Enterocloster bolteae* grows better in most MCBAs with CA core than unconjugated CA,^64^ although this was not the case for all MCBA producers. For example, Phe-CA decreased the growth of the MCBA producer *Lacrimispora indolis* while increasing the growth of another MCBA producer of *Lactiplantibacillus plantarum*, but the opposite was true of TCA.^64^ MCBA dependent inhibition was extended to a total of 18 strains and the inhibition of each species was dependent on the amino acid conjugated to CA *in vitro*.^64^ Of the MCBAs, Phe-CA and Leu-CA had the strongest levels of inhibition for the most species, stronger than TCA and GCA, indicating that MCBA generation increases the toxicity of bile acids.^64^ Generally, the greater the hydrophobicity of the conjugated amino acid in the MCBA the greater the antimicrobial properties.^64^ Species dependent inhibition by MCBAs was also confirmed *in vivo*. Mice fed the MCBAs Phe-CA and Ser-CA had significant shifts in the beta diversity of their fecal microbiota, though this effect was subtle and lost when comparing between groups at individual timepoints.^64^ Changes to the chemical structure of bile acids by gut microbes play an important role in shaping gut ecology at the species level and are an important factor when considering new therapies for CDI and other intestinal diseases.

## Bile acids and the host response

Bile acids not only interact with the microbiota but also with the host. These interactions are reviewed in Collins et al. 2023.^100^ During enterohepatic circulation, bile acids can directly interact with the host through nuclear receptors, such as FXR and PXR, which regulate the production of bile acids in the host. This can also influence other receptors such as RORγT that alters host physiology, such as immunity, by modulating T_reg_ and Th_17_ cell differentiation.^27,45,101–104^ TGR5 also interacts with bile acids.^103,105^ Among these, FXR and TGR5 are the only receptors whose known agonists are solely bile acids, and they both react differently depending on which bile acid is bound.^101,103^ The differential-binding ability of bile acids to host receptors has been reviewed by Fiorruci et al. 2021.^104^

FXR has differential activation depending on the bile acid it encounters in the order of CDCA>CA>LCA>DCA.^104,106^ Both murine and human FXR are activated by MCBAs, again with varying responses to each bile acid, the highest activation is with CDCA conjugates.^63^ Increased FXR signaling is associated with successful FMT, though FXR signaling does not appear to be significantly altered in patients with primary CDI.^32,45^

FXR activation regulates the production of bile acids by the host through controlling the expression of the gene that encodes the rate limiting enzyme of host bile acid synthesis: *Cyp7a1*.^101^ FXR may also modulate the nutritional environment in the gut, specifically the amount of taurine present through increase of host taurine synthesis and bile acid amidation.^107^ This plays an important role in modulating blood cholesterol levels and the amount of bile acid present in the gut. Diet can also modulate bile acid levels, for example dietary polyphenols modulate bile acid metabolism and signaling pathways.^108^ Not only is FXR important in regulating bile acid synthesis, but it has been shown to be important in modifying the immune response. FXR can activate the NLRP3 inflammasome in inflammatory macrophages, thereby modulating aspects of the innate immune system.^109^ FXR is also able to stabilize the transcriptional regulator, NCoR, on the NF-kB binding site of IL-1B, preventing its transcription and reducing the inflammatory response.^110^ Certain diseases, such as IBD, also have higher expression of FXR increasing sensitivity to fiber-induced changes to microbiota-derived bile acids such as increased CA and CDCA.^111^ Treatments targeted at modifying the microbiota to restore bile acid metabolism will also directly impact the host through receptors such as FXR.

Bile acids, while toxic to certain microbes such as *C. difficile*, can also be toxic to the host. PXR can be activated by various xenobiotics including pharmaceuticals, nutraceuticals, dietary factors, environmental chemicals, as well as secondary bile acids.^112^ PXR regulates the gut liver axis through bidirectional interactions with the gut microbiome under different conditions, to reduce the amount of toxic bile acids. MCBAs, particularly those conjugated to glutamate, have been observed to interact with PXR in mouse organoids and human cell culture reporter models.^63^

RORγT is particularly interesting with regard to CDI as this bile acid receptor modulates T cells and is activated differently depending on which bile acid is present.^113^ The microbial-derived bile acid isoDCA promotes the generation of colonic FOXP3 regulatory T cells expressing RORγT through interaction with dendritic cells.^113^ RORγT also plays a role in the differentiation of Th_17_ and T_reg_ cells in response to microbially derived bile acids.^27^ Diet also plays a role in RORγT signaling.^102^ In mice, a change in diet due to weaning alters RORγT signaling and was associated with an increase in Clostridia, a class of organisms rich in 7α-dehydroxylation of bile acids.^114^ Diet is also important as malnutrition has also been observed to increase the number of cells expressing RORγT.^115^ Increases in RORγT cells have been observed in mouse models of FMT for IBD.^116^

Leveraging the microbiota to alter bile acids to modulate nuclear receptors may be able to impact inflammation and immunity in patients with CDI. However, it is essential to acknowledge that bile acids are not always beneficial to the host. Certain bile acids or an excess of bile acid such as CDCA, DCA, and LCA have been observed to increase inflammation in the gut, so it will be important to be able to control the concentration delivered.^117–119^ Inflammation, including that resulting from CDI, has wide reaching impacts including increasing host-derived microbial nutrients in the gut, impacting the colonization of microbes, and affecting the metabolome.^119,120^

## Non-bile acid dependent microbial interactions and *C. difficile*

Cross feeding, or the production of essential nutrients for *C. difficile* by gut microbes, is important to consider when studying how *C. difficile* establishes colonization. Amino acids are some of the most important nutrients to consider in cross-feeding, as *C. difficile* is auxotrophic for six amino acids: cysteine, isoleucine, leucine, proline, tryptophan, and valine.^121^ Most cases of cross feeding involve *C. difficile* using nutrients produced by other gut microbes while generating minimal nutrients in return.^122^ *In silico* models predict that *C. difficile* consumes amino acids produced by commensal organisms such as *Bifidobacterium* longum, including proline, glutamate, leucine, tyrosine, alanine, serine, glutamine, and methionine while only providing aspartate in return.^122^ Enterococci cross feeds *C. difficile* with amino acids including leucine and ornithine, resulting in a high *C. difficile* load and worse disease in a mouse model of CDI.^33^ These findings were mirrored in CDI patients colonized with vancomycin-resistant *Enterococcus* (VRE).^33^ While Enterococci is involved in cross feeding, it can also limit the amino acids available to *C. difficile*. Competition for arginine between *Enterococcus faecalis* OG1RF and *C. difficile* causes stress in *C. difficile*, which in turn increases virulence factor expression and promotes disease.^33^ *C. sardiniense* also cross feeds *C. difficile* with amino acids including ornithine, leading to an increase in pathogen biomass and disease in a mouse model.^123^

Host factors also play an important role in nutrient-mediated support of *C. difficile*. The gut environment of pre-FMT rCDI patients contains higher levels of host-associated acylcarnitines, which other members of the gut microbiota can use for growth.^124^ This suggests that *C. difficile* may be engaging in cross-feeding by liberating host molecules via toxin production.^44,124^ Hydroxyproline is the main ingredient of collagen and is an amino acid-rich nutrient that *C. difficile* likely liberates from the host via toxin mediated inflammation. Toxin mediated inflammation disrupted collagen networks, which supported *C. difficile* growth.^120^ Hydroxyproline is converted to proline, a key nutrient for *C. difficile*, by *hypD* and *proC*, genes widely found in many commensals and in *C. difficile*.^125^ The presence of hydroxyproline affects the metabolic gene expression of both *C. difficile* and commensal Clostridia strains including *C. scindens* VPI 12708, *C. hylemonae* TN 271, and *C. hiranonis* TO 931, suggesting that it influences nutrient competition and adaptation within the gut environment.^125^ Specifically, proline reductase genes are upregulated in *C. difficile*, while hydroxyproline is present *in vitro*.^125^ This increase in proline reductase genes was not consistent across the commensal species tested indicating hydroxyproline may be preferred by specific species.^125^ A mouse model comparing wild type *C. difficile* to *hypD* knockout *C. difficile* noted changes in the microbiota, mainly as an increase in Lachnospiraceae.^125^

Nutrient competition between members of the gut microbiota and *C. difficile* is another mechanism by which colonization resistance can be maintained. Gut communities with members that compete with pathogens for key nutrients have been shown to restore colonization resistance against *Klebsiella pneumoniae* and *Salmonella enterica* serovar Typhimurium in a mouse model.^126^ Since *C. difficile* and many other Bacillota use Stickland fermentation to generate energy, substrates such as glycine, isoleucine, leucine, proline, and hydroxyproline are often the cause of competition between microbes.^121,127–129^ In a mouse model of CDI, competition for Stickland metabolites between *C. difficile* and commensals like *C. hiranonis* 10542, *Clostridium leptum* ATCC 29065, or *C. scindens* VPI 12708 is enough to prevent CDI-related weight loss.^32^ As these organisms encode a *bai* operon, which may be producing inhibitory bile acids, the ability for these organisms to compete with *C. difficile* and prevent CDI-related weight loss was validated in a knockout mouse that does not produce CA.^32^ There was also an increase in Stickland metabolites, namely 5-aminovalerate which is a byproduct of proline fermentation, in germfree mice monoassociated with the commensal strains.^32^ In another study, monocolonization of germ-free mice with the *C. scindens* ATCC 35704 prior to *C. difficile* challenge delayed clinical signs of disease and colonic damage for a few weeks, and did not prevent CDI.^130^ In gnotobiotic mice with a defined consortium, *C. scindens* ATCC 35704 also did not prevent colonization of *C. difficile*.^130,131^

While these strains contain the *bai* operon and, therefore, may produce inhibitory secondary bile acids, other strains such as *Paraclostridium bifermentans* that do not encode the *bai* operon also compete with *C. difficile*, and increases survival in a CDI mouse model, indicating that competition for nutrients between *C. difficile* and its close relatives may prevent or reduce disease.^32,123^

Non-toxigenic strains of *C. difficile* are also able to colonize the gut of rCDI patients and reduce CDI recurrence, likely by competing for the same nutrients.^132^ Considering that many of these Bacillota are lost during antibiotic treatment, they are a population which therapies often seek to reestablish with the hope of increasing colonization resistance. For example, a strain of *C. scindens* ATCC 35704 produces an antimicrobial alkaloid derived from L-tryptophan and oxaloacetaldehyde, 1-acetyl-β-carboline, that inhibits *C. difficile*.^133^ Another microbial metabolite, Urolithin A, has been observed to reduce the expression of *C. difficile* toxin and repair epithelial damage.^134^ These metabolites and the bacteria that produce them are important to consider when designing novel therapeutics against *C. difficile*.

## Rational design and standardization of microbiota-focused therapeutics to combat rCDI

While FMT serves as a last resort in treating patients with rCDI, the lack of knowledge of specific mechanisms that mediate its success impedes the development of microbiota-focused therapeutics. Defining which bacteria are needed for a successful FMT is a challenge. We are still not sure if the ability of donor strains to inhabit the recipient after an FMT, a process called engraftment, is required for successful treatment. A recent meta-analysis found that the clinical success of FMTs for rCDI was associated with donor strain engraftment and convergence of microbial species abundance.^135^ Although this process is taxonomically biased, some taxa have more difficulty engrafting than others.^136^ However, engraftment is not always required for successful treatment with FMTs or LBPs. Some microbiota-focused therapeutics and LBPs used to treat rCDI do not always show complete engraftment observe increases in beneficial taxa even if they are not included in the formulation.^51,53^ Additionally, benefits from probiotic strains, such as lactobacilli, can be observed even in the absence of engraftment, as they are passing through, but still providing a function.^33^ The complexities of FMT, including the intricacies of engraftment and variable roles of individual bacterial taxa, underscore the need for further research to decipher the mechanisms behind its success.

Attempts to systematically reestablish the functions that are lost after antibiotic treatment with regard to CDI are ongoing. Rebyota (RBX2660) is a product produced by Ferring Pharmaceuticals that achieved FDA approval in 2022 and became the first microbiota-based therapeutic to enter the market. Rebyota is pathogen screened feces that has been filtered after suspension in a solution of saline and polyethylene glycol 3350 mix.^11,137,138^ Additional screens ensuring a minimal dose of *Bacteroides* and a maximum dose of polyethylene glycol 3350 were also performed.^11,137,138^ This product is similar to FMT as it utilizes minimal processing thereby keeping the microbiome of the donor mostly intact. Patients treated with Rebyota have similar changes to the bile acid pool as those observed in FMT studies, a reduction of primary bile acids with an increase in secondary bile acids (Table 1).^48,49^ While this is a critical first step in standardizing the process of introducing beneficial bacteria to treat rCDI, there are still potentially unnecessary bacteria in this preparation. VOWST, another microbiota-focused therapeutic previously known as SER-109, produced by SERES Therapeutics also attained FDA approval in 2023. VOWST is produced using an ethanol-based purification method to enrich spore forming cells, namely Bacillota.^10,51^ This method has a similar success rate of 88% for the treatment of rCDI compared to Rebyota 87.1% in their respective clinical trials.^50,52^ This is similar to the success rates reported for FMT which range from 81% to 93%.^6,46^ VOWST was able to restore the presence of secondary bile acids after treatment, similar to Rebyota and FMTs (Table 1).^51^ Patients treated with VOWST also had an increase in the amount of *Bacteroides*, which was absent from the treatment administered.^51^ This highlights that a successful microbiota-focused therapeutic does not necessarily have to contain all of the bacteria necessary to treat rCDI. The bacteria administered might play an important role in changing the existing gut ecology, which provides resistance against *C. difficile*.

While the trajectory of development for products like Rebyota or VOWST is a top-down approach starting with healthy donor feces, LBPs composed of defined consortia aim to build an effective set of microbes from the bottom up. The Nubiyota product, Microbial Ecosystem Therapeutic-2 (MET-2), is comprised of 40 strains of bacteria and has shown promise in phase 1 clinical trials to treat rCDI as well as phase 2 clinical trials for depression and general anxiety disorder.^12,139^ Similar to FMT and other LBPs, MET-2 is associated with an increase in alpha diversity post-treatment (Table 1).^12^ VE303, developed by Vedanta Biosciences, is a formulation of eight Clostridia strains. In a recent phase 2 trial, this consortia achieved a rCDI remission rate of 86.2% in the high-dose group, an outcome consistent with FMT and other microbiota-focused therapeutics.^13^ After treatment with VE303 in patients who recently received antibiotics there was an increase in deconjugated bile acids and secondary bile acids, as well as a decrease in conjugated primary bile acids, indicating a return of microbes encoding bile acid altering enzymes (Table 1).^53^ In a first of its kind dose–response study on vancomycin-treated patients receiving VE303, the microbiota recovered significantly quicker in high-dose cohorts (8.0 × 10^9^ CFU/day) vs low-dose cohorts (≤4.0 × 10^9^ CFU/day).^53^ A common theme among LBPs is smaller communities being biased toward Bacillota in which most 7α-dehydroxylation occurs.^90,140^

## Microbiota-focused therapies beyond rCDI

FMTs and LBPs have shown promise in other diseases outside of rCDI. Perhaps, the most direct translational diseases include other bowel and liver diseases such as IBD, including Ulcerative Colitis (UC) and Crohn’s disease (CD), and NAFLD that are all associated with shifts in the bile acid pool.^141,142^ The guidelines set by the American Gastroenterological Association (AGA) state FMTs are recommended for the treatment of rCDI in immunocompetent and mildly immunocompromised adults, while microbiota-based therapeutics such as Rebyota and VOWST are recommended for use only in immunocompetent adults, however, no microbiota-based therapeutic is recommended for IBD at this time.^7^ Understanding the overlap between rCDI and other intestinal diseases will aid in our understanding of how to best leverage microbiota-based therapeutics. IBD is marked by increases in primary bile acids such as CA and CDCA, alongside decreases in abundance of secondary bile acids such as DCA and LCA, similar to changes observed in rCDI.^44,141^ Changes to the serum bile acid pool in NAFLD also include an increase in primary bile acids and secondary bile acids as well as a decrease in conjugated bile acids overall in serum.^142^

Increased levels of secondary bile acids and SCFAs were found in UC patients who responded to FMT in a randomized trial.^143^ An open label clinical trial comparing gastroscopy and colonoscopy for delivery of FMTs to treat CD achieved 66.7% remission rates and increased the taxonomic diversity in the gut.^144^ In cases where CDI co-occurs with IBD, FMTs have been observed to have a positive effect on both diseases at the same time.^145^ Liver-related diseases such as NAFLD, including nonalcoholic fatty liver (NAFL) and nonalcoholic steatohepatitis (NASH) have shown promise with microbiota-focused therapies. A clinical trial using a consortium of eight probiotic strains, called VSL#3, including *Streptococcus thermophilus, Bifidobacterium breve*, two strains of *Bifidobacteria animalis, Lactobacillus acidophilus, Lactobacillus plantarum, Lactobacillus paracasei*, and *Lactobacillus helveticus* improved NAFLD.^146^ FMTs have also been evaluated for other diseases in which gut bacterial bile acid metabolism is also thought to be important, such as diabetes mellitus.^47^

The gut-brain axis is another active area of research in the context of bile acid metabolism. Bile acids share structural similarities with other cholesterol-derived hormones, and as such research in bile acid modifications may be applicable to these structurally similar molecules. One such instance is polycystic ovarian syndrome (PCOS), which is characterized by an excess of androgens.^147^ In studies using mice colonized with microbiomes from women with PCOS, it was observed that there was a disruption of ovarian function coupled with altered bile acid metabolism compared to mice colonized with a control microbiome.^147^ As mentioned previously, there are also links to depression, with gut encoded HSDHs being implicated, as well as MET-2 the defined consortia in clinical trials used to treat depression.^76,77,139^ A reduction in symptoms associated with autism spectrum disorder (ASD) has also been observed alongside changes in blood neurotransmitter levels in open label clinical trials.^148^ Validation of the open-label trials, ideally through double blinded randomized controlled trials will be critical in further evaluating the efficacy of FMT for these diseases. Other neurological disorders such as Alzheimer’s, stroke, epilepsy, Tourette Syndrome, diabetic neuropathy, and Guillain–Barre are also being investigated, although limited evidence for efficacy is currently available.^149^

## Conclusions and future directions

*C. difficile* is a major healthcare-associated pathogen with significant morbidity and mortality. CDI is associated with antibiotic usage, which disrupts the native gut microbiota, diminishing colonization resistance against *C. difficile*. A deeper understanding of the underlying mechanisms facilitating FMTs and LBPs, which aim to reinstate colonization resistance, will allow for targeted approaches to their development. We have reviewed *C. difficile*’s high sensitivity to bile acids, and the microbial modifications that enhance this susceptibility. These modifications, mediated by the gut microbiota through enzymes like BSHs, HSDHs, and those in the *bai* operon play a pivotal role in altering bile acid profiles. Moreover, these modifications not only influence *C. difficile*, but the composition of the microbiota and host responses as well. Beyond bile acid modifications, other mechanisms such as cross-feeding, nutrient blocking, and the production of antimicrobial metabolites warrant consideration for optimizing FMT and LBP therapies. Ongoing efforts in this optimization process include the FDA approved Rebyota, a product comprised pathogen screened feces, and VOWST, a product comprised feces enriched for spore-forming bacteria, as well as ongoing clinical trials using defined bacterial consortia such as VE303 and MET-2. These products have been shown to alter the gut microbial community as well as the bile acid pool reflecting changes after an FMT. FMTs and LBPs have demonstrated potential efficacy in treating various other diseases such as IBD including UC and CD, NAFLD, PCOS, ASD, Alzheimer’s, stroke, epilepsy, Tourette syndrome, diabetic neuropathy, and Guillain–Barre syndrome.

The diversity of modifications to the bile acid pool has exploded recently to include MCBAs and newer modifications are continuing to be discovered.^150^ Exploring the impact of these novel bile acids in the context of rCDI prevention and treatment is one of the newest uncharted territories, warranting the need for comprehensive studies. Host produced and microbial-derived bile acids play a critical role in inhibiting *C. difficile* spore germination, growth, and toxin production, and continued evaluation of each bile acid’s role in these processes will be critical in developing therapies for rCDI. By leveraging the different antimicrobial effects bile acids have on different members of the microbiota, new therapies have the potential to shape not only pathogen dynamics, but commensal dynamics. This highlights the need to not only evaluate how bile acids impact pathogens, but also commensal organisms in the gut. Alongside these changes to the microbiome, bile acids also interact with host receptors and can mediate immune responses, highlighting their importance in developing therapies for a variety of diseases. More research is needed to understand how MCBAs and other non-canonical modified bile acids interact with host receptors and the consequences this has for host immunity and the gut microbiota. Other than bile acid mediated interactions with *C. difficile*, the microbiome, and the host, competition for nutrients and cross-feeding in the gut is becoming an important mechanism to evaluate while developing microbiota-focused therapeutics. Investigating the intricate connections between these elements will be important for developing successful microbial-focused therapeutics.
