# Correction

**DOI:** 10.1080/19490976.2024.2411134

**Published:** 2024-10-03

**Article title**: Bile acids impact the microbiota, host, and C. difficile dynamics providing insight into mechanisms of efficacy of FMTs and microbiota-focused therapeutics

**Authors**: McMillan, A. S., & Theriot, C. M.

**Journal**: *KGMI: Gut Microbes*

**DOI**: https://doi.org/10.1080/19490976.2024.2393766

The article was originally published with the incorrect Figure 1.

**Figure 1.**
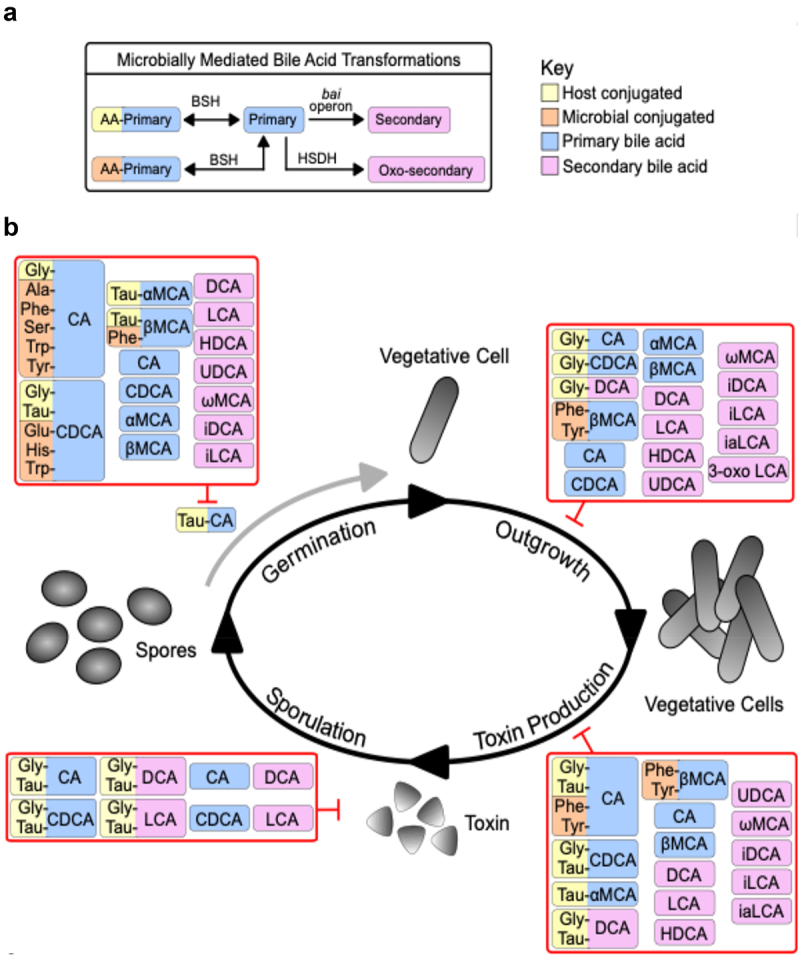

The correct version of figure 1 provided below and the text corrections have been included in the original article, and it has been republished accordingly.

